# Diversion Colitis and Probiotic Stimulation: Effects of Bowel Stimulation Prior to Ileostomy Closure

**DOI:** 10.3389/fmed.2021.654573

**Published:** 2021-06-25

**Authors:** Ángela Rodríguez-Padilla, Germán Morales-Martín, Rocío Pérez-Quintero, Ricardo Rada-Morgades, Juan Gómez-Salgado, Carlos Ruiz-Frutos

**Affiliations:** ^1^Department of General Surgery, Infanta Elena University Clinical Hospital, Huelva, Spain; ^2^Department of General Surgery, Juan Ramón Jiménez University Clinical Hospital, Huelva, Spain; ^3^Department of Sociology, Social Work and Public Health, Faculty of Labour Sciences, University of Huelva, Huelva, Spain; ^4^Safety and Health Postgraduate Programme, Universidad Espíritu Santo, Guayaquil, Ecuador

**Keywords:** diversion colitis, efferent loop stimulation, ileostomy closure, inflammatory bowel diseases, IBD management, probiotics

## Abstract

**Background:** Diversion colitis is a non-specific inflammation of a defunctionalised segment of the colon after a temporary stoma has been performed. This inflammation is associated with a change in the colonic flora.

**Aim:** To evaluate the efficacy and safety of preoperative stimulation of the efferent loop with probiotics prior to closure of the protective ileostomy in patients operated on colorectal carcinoma and its effect on diversion colitis. A prospective, randomised, double-blind, controlled study is carried out.

**Methods:** Patients who underwent surgery for colorectal carcinoma with protective ileostomy pending reconstructive surgery and with diversion colitis as diagnosis are included. Randomised and divided into two groups. Histological and endoscopic changes were evaluated after stimulation, after restorative surgery and during the short-term follow-up after surgery.

**Results:** Patients in CG were distributed according to the endoscopic index of severity in pre-stimulation/post-stimulation as follows: severe *n* = 9/9 (25.7%), moderate *n* = 23/23 (65.7%), and mild *n* = 3/3 (8.6%); compared to the distribution in SG, severe *n* = 9/0 (26.5/0%), moderate *n* = 23/3 (67.6/8.8%), mild *n* = 2/19 (5.9/55.9%) and normal colonoscopy in 0/12 patients (0/35.3%).

**Conclusion:** Probiotic stimulation of the efferent loop is a safe and effective method, managing to reduce both macroscopic and microscopic colitis, as well as a decrease in symptoms in the short term after reconstructive surgery.

## Introduction

In Western countries, rectal cancer represents 28–35% of all colorectal cancers, with an incidence of 15–25 new patients per 100,000 inhabitants/year. Its greatest complication is anastomotic leakage, with an incidence of 25% (1). The creation of a temporal ileostomy in patients operated on for colorectal carcinoma by means of a low anterior resection with total excision of the mesorectum, reduces the morbidity associated with anastomotic leakage (2). Around 18–40% of patients will present complications, such as diversion colitis, small bowel obstruction, surgical wound infection, postoperative ileus, anastomotic leak, fistula, perforation, abscess, bleeding, or hernia (3).

Diversion colitis (DC) is an inflammation produced in a defunctionalised segment of the colon after a temporary stoma has been performed (4). Described by Glotzer et al. (5), it is characterised by an inflammation of the large bowel mucosa that mimics idiopathic inflammatory bowel disease (4). It can manifest symptoms such as abdominal or pelvic pain, mucous discharge, tenesmus, fever, and rectal bleeding in the most severe cases, although up to 30% of patients remain asymptomatic (6). There are several hypotheses to explain the pathogenesis. One of them relates chronic inflammation with a decrease of bacteria in the dysfunctional area (7, 8). This inflammation causes endoscopic findings such as mucosal friability, oedema, erythema, appearance of polyps, ulcers, stenosis, and microscopic findings such as lymphoid follicular hyperplasia, infiltration of the lamina propria by lymphocytes, eosinophils, the appearance of plasma cells, architectural disruption, and the appearance of crypt abscesses (4).

The definitive treatment is the restoration of the continuity of the digestive tract (4, 6). Pharmacological treatments using instillations with short-chain fatty acids, mesalazine fibre, or corticosteroids are reserved for patients who are not candidates for surgical treatment or for stimulation of the efferent loop prior to surgery (9, 10). Stimulation with probiotics prior to closing the protective stoma would allow the dysfunctional colon segment to be repopulated, which would reduce diversionary colitis (11).

Probiotics are defined as live microorganisms that, when administered in adequate amounts, confer a health benefit on the host (12, 13). These and their metabolic products have been proposed as food supplements to achieve a healthier intestinal homeostasis and also as a treatment for pathologies with an important inflammatory component. Probiotics interact with the intestinal mucosa, reducing the molecular production of pro-inflammatory substances (12, 14). This immunomodulatory effect is what is needed in order to reduce the level of diversion colitis. Currently available probiotics, aimed at other pathologies with inflammatory conditions, produce a modulating effect in a transitory and limited way. This time-limited effect is what is needed to reduce the level of diversion colitis, since after reconstruction this tends to disappear.

## Materials and Methods

### Study Design

Prospective, randomised, multicentre, double-blind experimental study, comparing two groups of patients operated on for colorectal carcinoma with protective ileostomy. One group includes patients treated with stimulation of the efferent loop with probiotics prior to transit reconstruction surgery; the other control group is stimulated without giving any substance.

### Simple Size

The sample size was calculated according to the incidence of diversion colitis published in systematic reviews. Assumed reduction in PI was 50% (100–50%). With a loss adjustment of 15%, it was necessary to have 30 patients per group. We recruited 34 patients in stimulated group and 35 patients in control group for a statistical level of 95% and a power of 0.8.

### Selection of Patients

**Figure 1:**
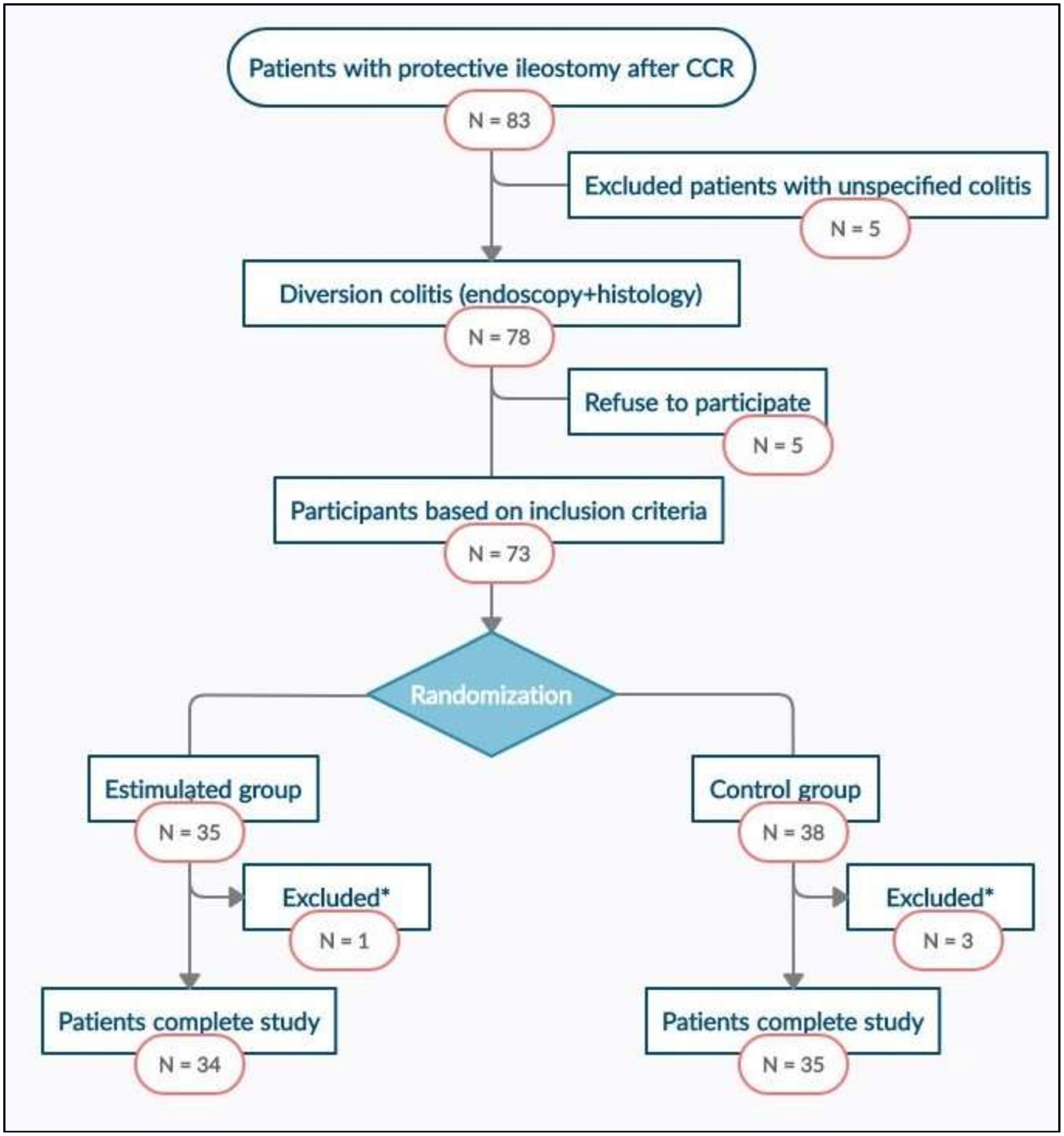
Study flowchart. CRC, Colorrectal cancer. *Excluded patients with anastomic leak.

Between January 2017 and December 2018, all the patients from participating centres included in the surgical waiting list for temporary stoma closure after colorectal carcinoma were consecutively evaluated to determine their inclusion in the study. The selection flowchart of the study patients can be seen in Figure 1.

The inclusion criteria were being over 18 years of age, having protective ileostomy after colorectal carcinoma surgery free of disease, with endoscopic and histological confirmation of diversion colitis and having signed the informed consent. The exclusion criteria being under 18 years of age, clinical history and histological confirmation of inflammatory bowel disease with colorectal involvement and refusal to participate in the study. Abandonment criteria: loss during follow-up, exitus, and anastomotic leakage after stoma closure.

### Randomisation and Intervention

A colonoscopy including biopsies for histological study, was performed on all patients selected to participate in the study. After confirming the diagnosis, level of colitis (based on the Harig scoring system) (15), and excluding patients who did not meet the selection criteria, randomisation was performed. Randomisation was performed by using a computer-generated sequence (Statistical software EPIDAT 4.1) into 2 groups:

- Stimulation group (SG): preoperative stimulation of the distal limb of the ileostomy loop with probiotics was performed during the 20 days prior to surgery every second day. During the process and after it, the patient registers the appearance of symptoms after each stimulation session: abdominal pain, emission of gas, and stool. A sterile Foley catheter No.14 Ch connected to an infusion set was introduced through the de-functioned bowel to allow the slow infusion of a solution with 4.5 mg of probiotics diluted in 250 ml of 0.9% physiological saline for 20–30 min. Each preparation was made under sterile conditions and maintaining the cold chain. Vivomixx® lyophilised live bacteria, marketed by MENDES, S.A, contained 4.5 × 10 11 of live bacteria in each preparation:
  ° Four strains of Lactobacillus:
    ▪ Lactobacillus acidophilus DSM 24735®
    ▪ Lactobacillus plantarum DSM 24730®
    ▪ Lactobacillus paracasei DSM 24733®
    ▪ Lactobacillus delbrueckii subsp. bulgaricus DSM 24734®
  ° Three strains of Bifidobacterium:
    ▪ Bifidobacterium breve DSM 24732®
    ▪ Bifidobacterium longum DSM 24736®
    ▪ Bifidobacterium infantis DSM 24737®
  ° One strain of Streptococcus
    ▪ Streptococcus thermophilus DSM 24731®
- Control group (CG): exactly the same procedure was carried out, but with the infusion set closed. During the process and after it, the patient himself registers the appearance of symptoms after each stimulation session: abdominal pain, emission of gas, and stool.

After ten stimulation sessions, 24 h before surgery, a colonoscopy with biopsy was performed on all patients, re-quantifying the endoscopic and histological index of severity of diversion colitis.

### Surgery and Follow-Up

All patients were admitted in the hospital the day before surgery, fasting, receiving antithrombotic prophylaxis (enoxaparin 40 mg subcutaneous) and premedication according to the pre-anaesthesia's instructions sheet. The reconstruction surgery was carried out by three expert surgeons from the Colorectal Surgery department. A parastomal incision was made and carried out sharply into the peritoneal cavity. The anastomosis was lateral-lateral, either manual or mechanical, according to the decision of the surgeon. Every surgeon was allowed to decide as well whether to change to a median laparotomy procedure. Complications or events happened during surgery were recorded in the surgical procedure protocol. General anaesthesia was given to all patients and, after extubation and stabilisation in the postoperative resuscitation room, they went directly to the hospitalisation ward.

Follow-up during hospitalisation was carried out by the staff of the Colorectal Surgery department of each centre, recording any postoperative complications, with special vigilance of abdominal pain, passage of flatus, or stool with correct quantification and initiation of oral tolerance. Patients were discharged from the hospital after re-establishing intestinal transit, adequate oral tolerance and stool control, recording the length of stay in hospital.

Follow-up after hospitalisation was carried out by Colorectal Surgery team in the first, third, and sixth postoperative months. These evaluations were performed by the colorectal surgeon who intervened in each patient. Any symptomatology related to the intervention was recorded, with special monitoring of abdominal pain and number and control of stools.

### Blinding

To ensure blinding of the patients, all underwent the same diagnostic procedure. During the stimulation sessions, both the solution with probiotics and the infusion set were covered by an opaque protective envelope, which prevented observing the colour and transparency of the fluid, or whether the system was open or closed. The stimulation sessions were performed by a single surgeon, who was also in charge of preparing the dilution.

The endoscopist, the pathologist and the surgeon who performed the surgical intervention and the follow-up, as well as surgeons who participated, after the surgery, in the hospitalisation process, did not know whether the patient had received probiotics or not.

### Assessment Criteria

The main evaluation criterion was the effect caused by stimulation of the efferent loop with probiotics on the persistence and severity index of diversion colitis, both endoscopic and histological, comparing SG and CG after stimulation, after surgery, in the first post-operative month and during short-term follow-up (third and sixth postoperative month).

Secondary evaluation criteria were passage of gas and stool during the stimulation period and after surgery, the initiation of oral tolerance, restoration of intestinal transit (gas and stool), and hospital stay.

### Statistical Analysis

A descriptive univariate analysis of sociodemographic and clinical variables was performed. The Kolmogorov-Smirnov test was used to verify the normality of the quantitative variables. To describe the quantitative variables, the mean and standard deviation were used, and the median and interquartile range for those variables that did not follow a normal distribution. For qualitative variables frequencies and percentages will be used. Afterwards, to verify the main objectives, a bivariate analysis was performed. A contrast test of proportions based on the Chi-square test was used in order to determine whether stimulation of the efferent loop with probiotics prior to the closure of the protective ileostomy reduces the level of diversion colitis, as well as its effects on the passage of gas and stool, the initiation of oral tolerance, re-establishing intestinal transit and hospital stay. To quantify its level of association, Cramer's Phi and V coefficients were calculated. In order to correlate quantitative variables, the Spearman's rank correlation coefficient was used. A *p* < 0.05 was considered to be significant. Statistical analysis was performed using the statistical program SPSS version 24.0, with the support of calculation tools provided by the software Microsoft Excel or R.

### Ethical Aspects

The project was performed with the consent of the Ethics Coordinating Committee for Biomedical Research of Andalusia, Spain, and registered with the project number 2017/331191354. Written informed consent was requested to participate in the study, giving details of both the study objectives and the methodology to be followed. The data was kept anonymous, maintaining the confidentiality and anonymity of the participants. The CONSORT Statement criteria for the design of clinical trial were followed (standard trial methodology).

## Results

### Study Population

Between January 2017 and December 2018, 83 disease free patients with protective ileostomy after colorectal carcinoma resection were reviewed and included in the surgical waiting list for intestinal transit reconstruction. Seventy eight of them met the endoscopic and histological criteria for diversion colitis diagnosis and 73 patients were finally randomised into two groups, intervention (*n* = 35) and control (*n* = 38). Sixty nine patients completed the study, 1 of them from SG and 3 from CG abandoning the study because of anastomotic leakage. Study flowchart is presented in Figure 1. There were no significant differences between SG and CG in terms of sociodemographic, clinical, or surgical variables (Table 1).

**Table 1:** Demographics, clinics, and surgical characteristics.

	**Stimulated group**	**Non-stimulated group**	***p***
	**(*n* = 34)**	**(*n* = 35)**	
**Demographics**			
Age (years)	65 (45 – 81)	68 (41 – 80)	0.130
Sex ratio (Men/Women)	23:11	25:10	0.733
BMI (kg/m^2^)	23.5 (21.6 – 32.6)	27.6 (18.8 – 40.2)	0.091
ASA			0.483
ASA I-II	31	30	
ASA III	3	5	
Smoker / Non-smoker	20/14	23/12	0.826
**Colorectal surgery**			
*Surgical procedure:*			0.129
LAR	25	25	
uLAR	9	10	
Surgical approach:			0.551
Laparoscopic	24	24	
Open	10	11	
Type of anastomosis:			0.430
Stapled EEA	31	33	
Coloanal anastomosis	3	2	
Neoadjuvant therapy	26	25	0.239
Adjuvant treatment	26	28	0.256
**Ileostomy closure**			
Time between surgery	12 (8 – 37)	9 (6 – 32)	0.813
*Surgery:*			0.690
Small bowel resection	33	34	
Ileocecal resection	1	1	
Surgical approach:			0.291
Peri-ileostomy	30	28	
Midline laparotomy	4	7	
Type of anastomosis:			0.355
Sewn	29	29	
Stapled	5	6	
Time (minutes)	50 (30 – 70)	65 (50 – 120)	0.053

*ASA, American Society of Anaesthesiologists Classification; BMI, body mass index; EEA, end to end anastomosis stapler; LAR/uLAR, low anterior resection/ultralow anterior resection*.

### Main Assessment Criteria

**Figure 2:**
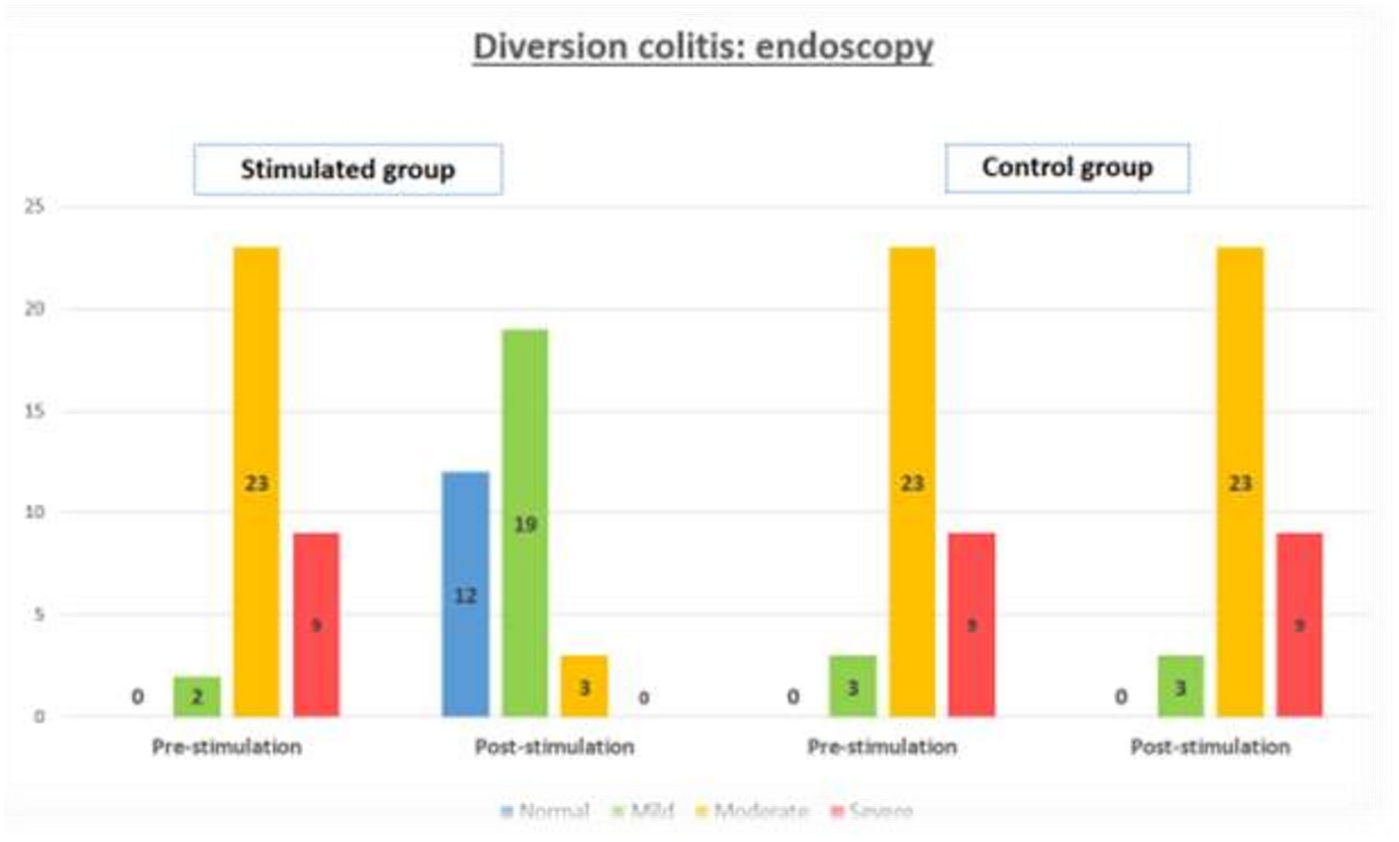
Grade of macroscopic diversion colitis, measured by endoscopy, in the stimulated group and control group, in the pre-stimulation and post-stimulation phases.

The effect of stimulation with probiotics on diversion colitis, identified by endoscopic, is showed in Figure 2. It shows a homogeneous distribution in the pre-stimulation phase between SG and CG (*p* = 0.911), with *n* = 9 patients with severe diversion colitis in both groups (26.5% SG vs. 25.7% CG), *n* = 23 patients with moderate diversion colitis in both groups (67.6% SG vs. 65.7% CG) and *n* = 2 patients with mild diversion colitis in SG (5.9%) vs. *n* = 3 patients in CG (8.6%). In the post-stimulation phase, CG maintains its distribution with *n* = 9 patients with severe diversion colitis (25.7%), *n* = 23 patients with moderate diversion colitis (65.7% CG), and *n* = 3 patients with mild diversion colitis (8.6%); on the other hand, SG shows no patients with severe diversion colitis (*n* = 0), *n* = 3 patients with moderate diversion colitis (8.8% CG), *n* = 19 patients with mild diversion colitis (55.9%) and *n* = 12 patients with normal endoscopic findings (35.3%), achieving a *p* < 0.001 and a Phi and V Cramer coefficient of 0.883.

**Figure 3:**
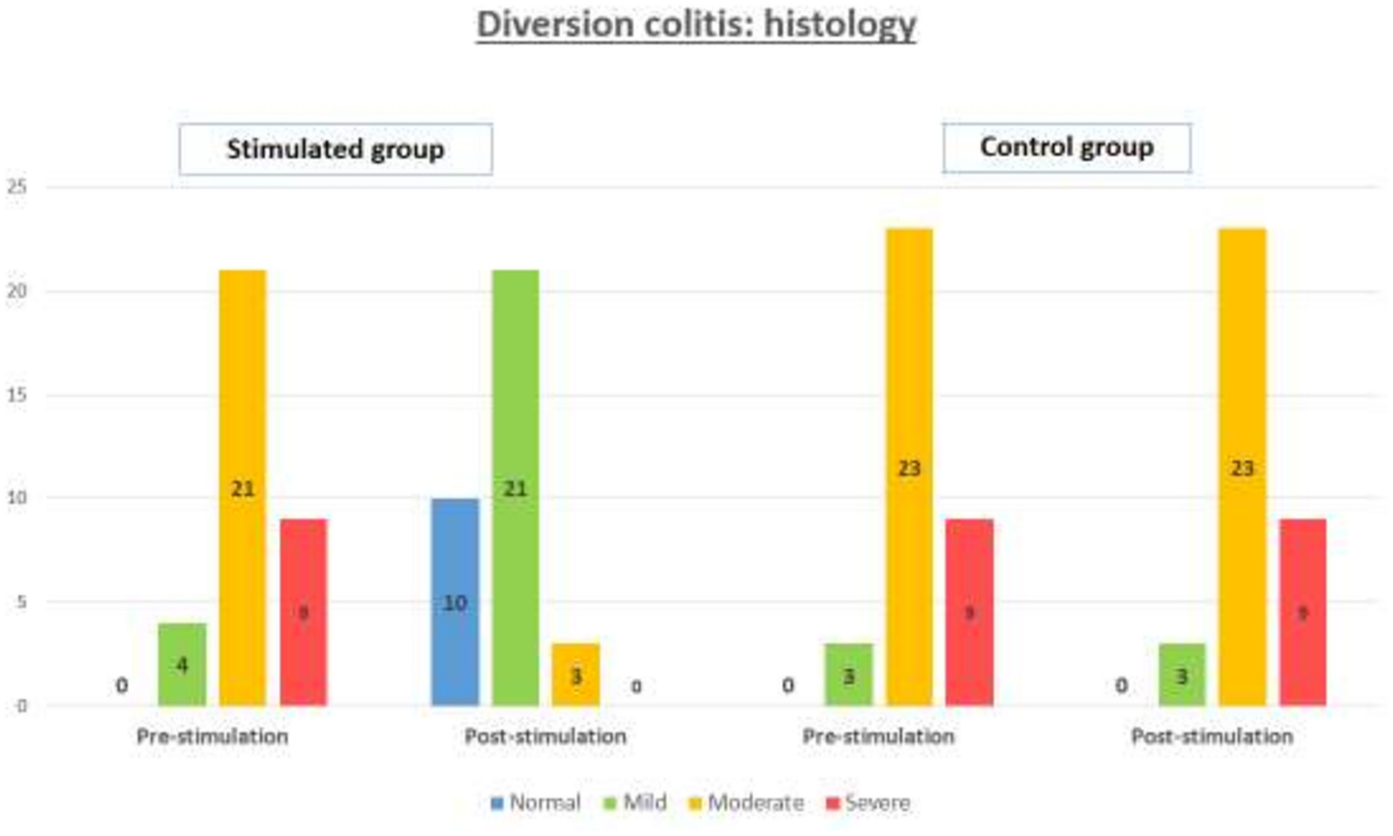
Grade of microscopic diversion colitis, measured by histology, in the stimulated group and control group, in the pre-stimulation and post-stimulation phases.

The effect of stimulation with probiotics identified by histology is showed in Figure 3. It shows a homogeneous distribution in the pre-stimulation phase between SG and CG (*p* = 0.896), with *n* = 9 patients with severe diversion colitis in both groups (26.5% SG vs. 25.7% CG), *n* = 23 in CG and *n* = 21 patients with moderate diversion colitis in both groups (61.8% SG vs. 65.7 % CG) and *n* = 4 patients with mild diversion colitis in SG (11.7%) vs. *n* = 3 patients in CG (8.6%). In the post-stimulation phase, the CG maintains its distribution with *n* = 9 patients with severe diversion colitis (25.7%), *n* = 23 patients with moderate diversion colitis (65.7% CG), and *n* = 3 patients with mild diversion colitis (8.6%), on the other hand, SG shows no patients with severe diversion colitis (*n* = 0), *n* = 3 patients with moderate diversion colitis (8.8% CG), *n* = 21 patients with mild diversion colitis (61.8%) and *n* = 10 patients with normal histologic findings (29.4%), getting a *p* < 0.001 and a Phi and V Cramer coefficient of 0.843.

Abdominal pain was the only symptom observed during stimulation, which appeared in 20.5% of patients SG (*n* = 7) compared to 14, 3% CG (*n* = 5). There were no statistically significant differences between the two groups. Abdominal pain was evaluated using the visual analogue scale (VAS). Pain was moderated in SG, only present in the first stimulation sessions and disappearing afterwards. Pain was mild-moderate in CG in all stimulation sessions and disappeared after their completion.

The symptoms of diversion colitis after reconstruction surgery appeared in 100% (*n* = 35) of the patients in CG, vs. 23.5% in SG (*n* = 8), the remaining 76.5% being asymptomatic after surgery (*n* = 26), with *p* < 0.001 and a Phi and V Cramer coefficient 0.789. This clinical scenario is maintained 1 month after reconstructive surgery in CG, with 94.3% (*n* = 33) of patients showing symptoms of diversion colitis, compared to 5.7% who are asymptomatic (*n* = 2). SG shows 97.1% of asymptomatic patients after surgery (*n* = 33) compared to 2.9% of patients with diversion colitis (*n* = 1), with *p* < 0.001 and a Phi and V coefficient of Cramer 0.913. Finally, during the short-term follow-up after reconstructive surgery, symptoms are present in CG with 28.6% (*n* = 10) compared to 71.4% who are asymptomatic (*n* = 25). SG shows 100% asymptomatic patients after surgery, with *p* < 0.001 and a Phi and V Cramer coefficient of 0.406.

### Secondary Endpoints

In the comparative analysis, we found a direct relationship between passage of gas and stool during the stimulation period and the decrease in the level of diversion colitis in SG with *p* < 0.001 and a Phi and V Cramer coefficient of 0.683 for stool and 0.971 for gas. There were no statistically significant differences among the initiation of oral tolerance, restoration of intestinal transit, or length of hospital stay, as shown in Table 2. The incidence of postoperative ileus was similar in both groups, appearing in 10/34 (29, 4%) for SG and 11/35 (31.4%) for CG with *p* = 0.192. After reconstructive surgery, SG started oral tolerance 24 h earlier than CG, but it was statistically non-significant (2 days vs. 3). The emission of gases and the restoration of intestinal transit happened 2 days after surgery in both groups. SG had an interval between 1 and 20 days in the emission of gases and between 1 and 21 days in the intestinal transit, vs. 1–48 days in both cases in CG. The hospital stay was also shorter in the SG, but without statistical significance.

**Table 2:** Postoperative results.

	**Stimulated group**	**Non-stimulated group**	***p***
	**(*n* = 34)**	**(*n* = 35)**	
Postoperative ileus, *n* (%)	10 (29.4%)	11 (31.4%)	0.192
Nasogastric tube, n (%)	9 (26%)	11 (31.4%)	0.116
Time to tolerating a diet, days-mean (range)	2 (1 – 24)	3 (2 – 50)	0.619
Start of the passage of flatus, days-mean (range)	2 (1 – 20)	2 (1 – 48)	0.173
Start of the passage of stool, days-mean (range)	3 (1 – 21)	3 (1 – 48)	0.184
Postoperative stay days-mean (range)	4 (4 – 26)	5 (4 – 56)	0.105

*The median has been used as a measure of central tendency for the evaluation of variables as initiation of oral tolerance, gas emission, restoration of transit and duration of hospital stay*.

## Discussion

As mentioned in the introduction, colorectal cancer surgery has progressively evolved. More and more complex resections are performed both by abdominal and perineal approach, increasing their quality and achieving a more radical surgery with increasingly inferior anastomoses and without the need for a permanent stoma, even if still requiring a temporary stoma (16). To protect low colorectal anastomosis from possible complications, we must create an ileostomy, which also will lead to morbidity. Derivative stomas are not exempt from complications, the most feared being the anastomotic leak however the most common is diversion colitis (2, 3, 17). In patients that require a protective ileostomy, dysbiosis happens in the excluded colon segment that leads to diversion colitis (6). This dysbiosis activates metabolic products that produce an immune response, causing an attack on the colonic mucosa and affecting the function of the regulatory immune cells that normally promote its homeostasis (18). This causes structural changes, such as atrophy of villi in the defunctionalised limb, and in consequence produce loss of smooth muscle area and reduced isometric contractility with loss of the intestinal absorption capacity (7). Intestinal dysbiosis has been linked to the pathogenesis of numerous chronic diseases, such as inflammatory bowel disease, and has been studied in order to identify its correction, with variable results (19, 20).

The bibliography about diversion colitis and its relationship with microbiota alteration is not extensive. In the last 2 years, two studies have been published about intestinal microbiota (19, 21); to which we must add, on the one hand, the latest systematic review on diversion colitis published by Kabir et al. (6) that reviews a total of 3,305 articles, eventually including 35 studies, and analyses the pathophysiology, clinical presentation and treatment of diversion colitis which concludes that there is a great variability of forms of presentation and a single definitive treatment, which is the reconstruction of the transit. Results on preoperative stimulation prior to the closure of the protective ileostomy published by Rombey et al. (10), including 8 studies with a total of 267 patients, despite the promising initial results, show that there is no sufficient quality evidence to recommend routine implementation of preoperative bowel stimulation in clinical practise. That is why we propose that microbial manipulation by stimulating probiotics prior to closing the protective ileostomy would allow the altered autoimmune function to be normalised, restore homeostasis, and reduce mucosal inflammation.

After completing the stimulation phase with probiotics, no statistically significant differences were detected in terms of sociodemographic, clinical or surgical variables. On the contrary, there is indeed a notable decrease in surgical time in SG, which we associate with the thickening of the distal end caused by stimulation and which facilitates the performance of the anastomosis. In the pre-stimulation phase, no differences were found in terms of index of severity in both groups, either by endoscopy or histology. After stimulation, a decrease of the index of severity was observed in SG compared to CG, both by endoscopy and histology, as shown in Figures 2, 3.

A reduction in the level of diversion colitis was achieved by 100% of SG patients, with the disappearance of macroscopic diversion colitis in 35.3%, obtaining normal endoscopic findings in 12 patients (*p* < 0.001 and a Phi and V Cramer coefficient of 0.883) and the disappearance of microscopic sign in 29.4%, obtaining normal histological findings in 10 patients (*p* < 0.001 and a Phi and V Cramer coefficient of 0.843). Both cases showed a strong correlation between stimulation with probiotics and the decrease of diversion colitis, with the appearance of colicky abdominal pain in 20.5% of the patients (*n* = 7) as the only side-effect, valued as moderate pain using the visual analogue scale (VAS) and only associated with the first stimulation sessions, disappearing afterwards. This effect has already been described in studies with stimulation of the efferent loop with short chain fatty acids (6, 10, 15).

At the same time as stimulation, an increase in stool and gas emission was observed during this phase in SG compared to CG, which we directly relate to a decrease or disappearance of diversion colitis after stimulation with probiotics with a value of *p* < 0.001 and a Phi and V Cramer coefficient of 0.683 for stool and 0.971 for gas, both showing the association with a strong correlation. Initiation of intestinal transit prior to reconstruction allows the identification of functional obstructions distal to the ileostomy that can cause mechanical ileus after surgery and that can be solved by endoscopic dilations or, on the contrary, it allows the identification of a level of incontinence associated with the anterior resection syndrome, which allows for early toilet training (22, 23).

Regarding the effect on bowel function after transit reconstruction, no differences were observed between the two groups, with a non-statistically significant time to recovery of transit and oral tolerance. The difference in terms of hospital stay was 1 day, with a median of 4 days in SG and 5 days in CG, associating it with 24-h difference in oral tolerance between SG, in which 58% of patients tolerate liquid diet in the first 24–48 h, compared to the CG, which requires 48–72 h. There were no differences in postoperative ileus between SG (*n* = 10, 29.4%) and CG (*n* = 11, 31.4%), highlighting a disagreement with systematic reviews published by Rombey and Garfinkle and other studies on post-operative ileus that seem to improve after stimulation of the efferent loop prior to closure of the ileostomy (7, 23–28). Nonetheless, both conclude with a lack of evidence to recommend the stimulation of the efferent loop in a routine way and emphasise the need to carry out multicentre studies with higher scientific quality, in order to verify their validity.

The observations presented here demonstrate that the decrease in endoscopic and histological findings associated with the decrease in inflammation means that stimulation of the efferent loop of the ileostomy with probiotics can be an alternative treatment in patients with symptomatic DC who are not candidates for reconstructive surgery as a treatment to resolve the colonic inflammation (29, 30). Finally, the stimulation of the efferent loop with probiotics can be an alternative treatment to resolve the inflammation in patients whose surgical option is not feasible.

The most studied probiotic bacterias are Bifidobacterium and Lactobacillus spp. These two produce a decrease of pro-inflammatory molecules and an increase of molecules that inhibit inflammation, also protecting against oxidative stress in humans and demonstrating an important role in intestinal disbiosis (7, 31, 32). Currently, the evidence shows that probiotics have a positive effect on systemic and oxidative inflammation, especially at the gastrointestinal level. L. acidophilus, one of the probiotics administered during our stimulation, has demonstrated its role in inflammatory regulation in multiple experimental studies (32–41). A randomised controlled clinical trial conducted by Jafarnejad et al. (42) describes the effect of supplementation with probiotics for 8 weeks, in this case, with VSL3 (a probiotic similar to the one administered in our study), on glycaemic status and inflammatory markers among pregnant women. The probiotic supplements contained eight strains of lactic acid bacteria (S.thermophilus, Bifidobacterium breve, Bifidobacterium longum, Bifidobacterium infantis, L. acidophilus, L. plantarum, L. paracasei, and L. delbrueckii subsp. Bulgaricus). They found a statistically significant decrease in TNF alpha and CRP levels in the probiotic group compared to the placebo. Results are superimposable to other studies (41).

Finally, it was possible to reduce diversion colitis symptoms after reconstructive surgery in 76.5% of SG (*n* = 26) with *p* < 0.001 and observing correlation between the absence of symptoms and stimulation with a V Cramer coefficient of 0.789, compared to CG, in which 100% of patients presented symptoms. This is maintained in the follow-up to the first month after closure ileostomy with 94.3% of symptomatic patients in CG vs. 2.9% in SG, with *p* < 0.001 and an association of 0.913. However, in the short-term follow-up, both tend to equalise, achieving 100% asymptomatic patients in SG and 71.4% in CG, with *p* < 0.001 and a Phi and V Cramer coefficient of 0.406. This correlation disappears between the third and sixth month, when diversion colitis becomes non-existent both endoscopically and microscopically. This fact coincides with the transitory and limited modulatory effect that probiotics produces on the intestinal mucosa (43–47) and with the recovery curve of intestinal function published in other studies (7, 9). However, as the main limitation of our study has been the sample size we cannot make definitive statements about the effectiveness of this technique. We will need additional multicentre studies on bowel function and preoperative stimulation prior to closure of the protective ileostomy to confirm these conclusions.

## Conclusion

The pathogenesis of diversion colitis is related with a chronic inflammation and decrement of flora in the dysfunctional area. The definitive treatment is the restoration of the continuity of the digestive tract, and the stimulation of the efferent loop with probiotics would allow patients to reduce complications in the pre/postoperative process. The stimulation with probiotics is a safe and feasible procedure with minimal adverse effects, being an option for patients who are not candidates for surgical treatment.

## Data Availability Statement

The original contributions presented in the study are included in the article/Supplementary Material, further inquiries can be directed to the corresponding authors.

## Ethics Statement

The project was performed with the consent of the Ethics Coordinating Committee for Biomedical Research of Andalusia, Spain, and registered with the project number 2017/331191354. The patients/participants provided their written informed consent to participate in this study.

## Author Contributions

ÁR-P, GM-M, RP-Q, RR-M, JG-S, and CR-F: conceptualisation, data curation, formal analysis, investigation, methodology, resources, writing—original draught, and writing—review and editing. ÁR-P, GM-M, and RP-Q: project administration. GM-M: software. RP-Q, JG-S, and CR-F: supervision. RR-M and JG-S: validation. GM-M, RP-Q, JG-S, and CR-F: visualisation. All authors contributed to the article and approved the submitted version.

## Conflict of Interest

The authors declare that the research was conducted in the absence of any commercial or financial relationships that could be construed as a potential conflict of interest.
